# Effect of simultaneous multislice imaging, slice properties, and repetition time on the measured magnetic resonance biexponential intravoxel incoherent motion in the liver

**DOI:** 10.1371/journal.pone.0306996

**Published:** 2024-08-09

**Authors:** Martin Loh, Tobit Führes, Christoph Stuprich, Thomas Benkert, Sebastian Bickelhaupt, Michael Uder, Frederik Bernd Laun

**Affiliations:** 1 Institute of Radiology, University Hospital Erlangen, Friedrich-Alexander-Universität Erlangen-Nürnberg (FAU), Erlangen, Germany; 2 MR Application Predevelopment, Siemens Healthcare GmbH, Erlangen, Germany; New York University Langone Health, UNITED STATES OF AMERICA

## Abstract

**Objectives:**

This study aims to investigate the previously reported dependency of intravoxel incoherent motion (IVIM) parameters on simultaneous multislice (SMS) acquisition and repetition time (TR). This includes the influence of slice thickness, slice gaps, and slice order on measured IVIM parameters.

**Materials and methods:**

Diffusion-weighted imaging (DWI) of the liver was performed on 10 healthy volunteers (aged 20–30 years) at 3T with a slice thickness of 5 mm, a slice gap of 5 mm, and a linear slice order. Diffusion-weighted images were acquired with 19 b-values (0–800 s/mm^2^) using both conventional slice excitation with an acceleration factor of one (AF1) and SMS excitation with an acceleration factor of three (AF3). Each of these measurements were carried out with two repetition times (TRs)– 1,300 ms (prefix s) and 4,500 ms (prefix l)–resulting in four different combinations: sAF1, sAF3, lAF1, and lAF3. Five volunteers underwent additional measurements using a 10 mm slice thickness and with AF1. Median signal values in the liver were used to determine the biexponential IVIM parameters. Statistical significances were assessed using the Kruskal-Wallis test, Wilcoxon signed-rank test, and Student’s t-test. In-silico investigations were also used to interpret the data.

**Results:**

There were no significant differences between the biexponential IVIM parameters acquired from sAF1, sAF3, lAF1, and lAF3. Median values of the perfusion fraction *f* were as follows: 29.9% (sAF1), 26.9% (sAF3), 28.1% (lAF1), and 27.5% (lAF3). In the 10 mm-thick slices, *f* decreased from 31.3% (lAF1) to 27.4% (sAF1) (*p* = 0.141).

**Conclusion:**

The slice excitation mode did not appear to have any significant influence on the biexponential IVIM parameters. However, our simulations, as well as values reported from previous publications, show that slice thickness, slice gaps, and slice order are relevant and should thus be reported in IVIM studies.

## Introduction

In conventional magnetic resonance diffusion-weighted imaging (DWI), tissue signal decays in good approximation in a monoexponential manner due to molecular water diffusion. In the case of diffusion weightings (*b*) within clinical ranges [1–3], a Gaussian approximation for diffusion is valid, and the apparent diffusion coefficient (*D*) could be derived by describing the diffusion signal *S*(*b*) with:

$$S\left(b\right)/S\left(0\right)=\text{exp}\left(-bD\right)$$ (1)

Additionally, the intravoxel incoherent motion (IVIM) effect can lead to a discrepancy between the monoexponential signal attenuation and the measured values at small b*-*values (at approximately *b* < 200 s/mm^2^) in certain organs, such as the liver [4–6]. This deviation is usually attributed to the presence of a perfusion compartment and, in most cases, is well approximated by a biexponential function (Eq 2):

$$S\left(b\right)/S\left(0\right)=\left(1-f\right)\text{exp}\left(-bD\right)+f\text{exp}\left(-bD^{*}\right)$$ (2)

The perfusion fraction *f* and the pseudodiffusion coefficient D* are properties of the additional perfusion compartment.

As IVIM imaging requires data from a large number of b*-*values [7–10], especially at lower ranges (< 200 s/mm^2^), measurement protocols are often long and arduous. Furthermore, DWI measurements intrinsically exhibit low signal-to-noise ratios (SNR) [11], which further increases the need for additional measurements for averaging purposes as well as longer acquisition times. Recently, the advent of simultaneous multislice imaging (SMS) has allowed for the simultaneous excitation of multiple slices [12], which has led to a notable reduction in scan times [13–16]. Several studies have already examined and exploited SMS for IVIM imaging applications [17–20]. Among these, two studies observed a noticeable change in *f* between conventional excitation and SMS, with acceleration factors of two or three. Specifically, Van et al. [18] and Tang et al. [17] found that *f* significantly decreased when the SMS technique was used.

However, it is important to note that these previous studies did not attempt to disentangle the effect of SMS from that of TR variations. Instead, they exploited the advantage of SMS—i.e., reduced TR and acquisition times—for higher SMS acceleration factors. While this methodology is reasonable in terms of demonstrating the advantages of SMS, it cannot be used to explain the nature of the observed IVIM parameter dependencies. Instead, a more prudent methodology would involve the comparison of data acquired using both SMS and non-SMS techniques under otherwise identical conditions.

In this study, we performed measurements using both SMS excitation as well as conventional imaging protocols with large and small TR to explore and distinguish between the effects of SMS and TR on the biexponential IVIM parameters of the liver. In-silico investigations were also performed, incorporating T_1_-effects as well as the inflow and outflow of blood magnetization to predict *f* values and comparing them to both our results as well as the results of previous studies.

## Materials and methods

### Volunteers

The study cohort consisted of 10 healthy volunteers with no known history of liver diseases. Volunteers were recruited between the 24^th^ of January and the 16^th^ of February 2023. Subjects were between 20 and 30 years old (mean age: 23.9 years; median age: 22.5 years) comprising four females and six males. The study was approved by the ethics committee of the Friedrich-Alexander-University Erlangen-Nürnberg and all volunteers were provided with a written declaration of informed consent.

### Acquisition parameters and protocol

Data were acquired at a 3T scanner (MAGNETOM Prisma, Siemens Healthcare, Erlangen, Germany) with a 32-channel spine array and an 18-channel body array. DWI was performed in the abdomen with a single-refocused diffusion-weighted echoplanar imaging research application sequence that allowed for the application of the SMS technique. RF pulses were sinc-shaped during, both the SMS and the non-SMS acquisition.

The selected b*-*values were adapted from the first 18 *b-*values in the optimized set proposed by Lemke et al. [7] for their “high perfusion regime, *b*_high_”. The maximum b-value measured was 800 s/mm^2^ to avoid kurtosis effects (c.f. [1–3, 21, 22] for more information on diffusional kurtosis), while a third average of b = 0 was used to ensure robust normalization; measurements were thus performed with a total of 19 b*-*values. For each b*-*value, diffusion gradients were applied in three directions: (1,0,0), (0,1,0), and (0,0,1) with respect to the scanner coordinate system. Table 1 lists all b*-*values used in this study as well as the first 18 b*-*values proposed by Lemke et al. [7] among with further acquisition parameters.

**Table 1:** MRI sequence parameters.

b-values (s/mm^2^)	0 (3×); 10 (3×); 20; 40; 70; 80; 130; 170; 190; 200; 250; 750; 800 (3×) (This study) 0 (2×); 10 (3×); 20; 40; 70; 80; 130; 170; 190; 200; 240; 740; 830; 980; 1,000 (Lemke at al. [7])
**TE (ms)**	65
**TR (ms)**	1,300; 4,500
**Slice thickness (mm)**	5; 10
**Slice gaps (mm)**	5; 15
**FOV (mm** ^**2**^ **)**	400 **×** 400
**Voxel size (mm** ^**2**^ **)**	4 **×** 4 interpolated to 2 **×** 2
**Phase partial Fourier factor**	6/8
**Echo spacing (ms)**	0.49
**Acquisition bandwidth (Hz/Px)**	2,942
**Fat saturation**	SPAIR
**Parallel imaging**	GRAPPA with acceleration factor 2 and 24 reference lines
**Phase direction**	Anterior-posterior

DWI was performed with a short TR of 1,300 ms for both conventional slice excitation (sAF1) as well as SMS excitation with an acceleration factor of three (sAF3). The same protocols were used to acquire data at a long TR (4,500 ms); henceforth referred to as lAF1 and lAF3, respectively.

In total, nine 5 mm-thick transversal slices were acquired in the liver during free breathing, with 5 mm slice gaps. This large slice gap was intentionally chosen to rule out slice cross-talk (also under free breathing conditions). Volunteers were instructed to breathe shallowly to reduce motion during image acquisition. The echo time (TE) was 65 ms and the field of view (FOV) was 400 **×** 400 mm^2^ with a matrix size of 100 **×** 100. The voxel size was interpolated by the scanner to a resolution of 2 **×** 2 mm^2^. Further acquisition parameters include a phase partial Fourier factor of 6/8, an echo spacing of 0.49 ms, and an acquisition bandwidth of 2,942 Hz/Px. Spectral-attenuated inversion recovery (SPAIR) was used for fat suppression. The parallel imaging method GRAPPA with an acceleration factor of 2 and 24 reference lines was applied to account for in-plane acceleration; an anterior-posterior phase direction was used. The following vendor-provided data correction algorithms were applied: “dynamic field correction” was used to correct for eddy current-induced distortion, “2D distortion correction” was used to correct for image distortions arising from gradient non-linearities, “pre-scan normalize” compensated for surface coil flare, and “raw filter” at “medium” strength was used to reduce Gibbs ringing.

Five of the volunteers underwent additional imaging with a slice thickness of 10 mm for sAF1 and lAF1 in order to further examine T_1_-effects and the influence of the slice thickness and slice gaps on biexponential IVIM parameters. The number of slices was reduced to four, while slice gaps were increased to 15 mm.

The total combined acquisition time for sAF1, sAF3, lAF1, and lAF3 was approximately 14 min. The acquisition time for the scan with 10 mm slice thickness was approximately 7 min.

### Data evaluation

Regions of interest (ROI) in the parenchyma-sparing major vessels of the liver were defined by a physicist (M. L.) using MITK v2021.10 (Medical Imaging Interaction Toolkit, German Cancer Research Center, Heidelberg, Germany) [23] on the *b* = 0 images for each separate slice and was copied for the remaining b*-*values. As in previous studies [24–26], we excluded major vessels from the evaluation as we wanted to focus on IVIM as a proxy for blood perfusion (instead of macroscopic flow). All images were checked for signal dropouts due to cardiac motion in the left liver lobe; if detected, the ROIs were adapted accordingly by excluding the left liver lobe on that particular slice. No co-registration of the images was performed. The segmentation was supervised by a board-certified radiologist (S. B.).

Further evaluation was performed using Python 3.9 using the nibabel (loading Nifti files), numpy (array operations), matplotlib (plotting), and scipy packages (curve fitting and statistical analysis). The ROIs were used to compute the median value over all voxels for each slice, b*-*value, and diffusion direction. The effect of large vessels on the averaged signal over the ROI is minimized by choosing the median signal instead of the mean. For each slice, all median signal values were normalized to the respective median signal value at *b* = 0. These signal values were subsequently arithmetically averaged over the same nominal b*-*values and each diffusion direction. These signal values were treated as data points for the final IVIM fitting routine for each volunteer and slice.

The IVIM fit was performed in a segmented manner in accordance with previous studies [27–33]. First, the monoexponential fit of *D* was used for b*-*values greater than or equal to 200 s/mm^2^, as the IVIM effect becomes negligible in this regime. The Levenberg–Marquardt fitting algorithm was used with a starting value of 0.9 μm^2^/ms for *D*.

The biexponential IVIM equation was then fitted to the data for all b*-*values to determine *f* and D*; *D* was held constant during this second step. The starting values used for *f* and D* during the fit were 25% and 10 μm^2^/ms, respectively. *f* and D* were also restricted to the following bounds: 0–50% and 0–10,000 μm^2^/ms, respectively. Furthermore, data points were weighted according to the number of averages acquired for those data points during the fitting: i.e., data collected at *b =* 0, 10 s/mm^2^, and 800 s/mm^2^ were given a weight of 3, while all other data points were equally weighted at weight 1.

### Statistical methods

The Shapiro-Wilk test was performed on each group of fitted IVIM parameters to test for normality. Depending on the results of the test, a one-way analysis of variance (ANOVA) or the Kruskal-Wallis test was performed on normally and non-normally distributed data, respectively, to test for any statistically significant differences between the different measurement setups used to acquire the 5 mm-thick slices. The short and long TR acquisitions using 10 mm-thick slices were compared using a Student’s t-test (if normally distributed) or the Wilcoxon signed-rank test (if not normally distributed). The level of significance was set to 0.05 for all tests.

### In-silico validation

We previously examined saturation effects in the context of T_1_-relaxation as well as inflow and outflow effects using a larger TR (3,600 ms) and developed an in-silico model to estimate saturation effects depending on factors such as blood flow velocity, slice thickness, and slice gaps [34]. The model accounted for changes in the position of blood between slices and slice gaps. The model was adapted to this study—with some enhancements—to determine whether it was consistent with the measurement results obtained.

The following section briefly outlines the numerical implementation of the model. Please refer to the supporting information in Loh et al. [34] for a more comprehensive description of the physical background of the model.

**Fig 1:**
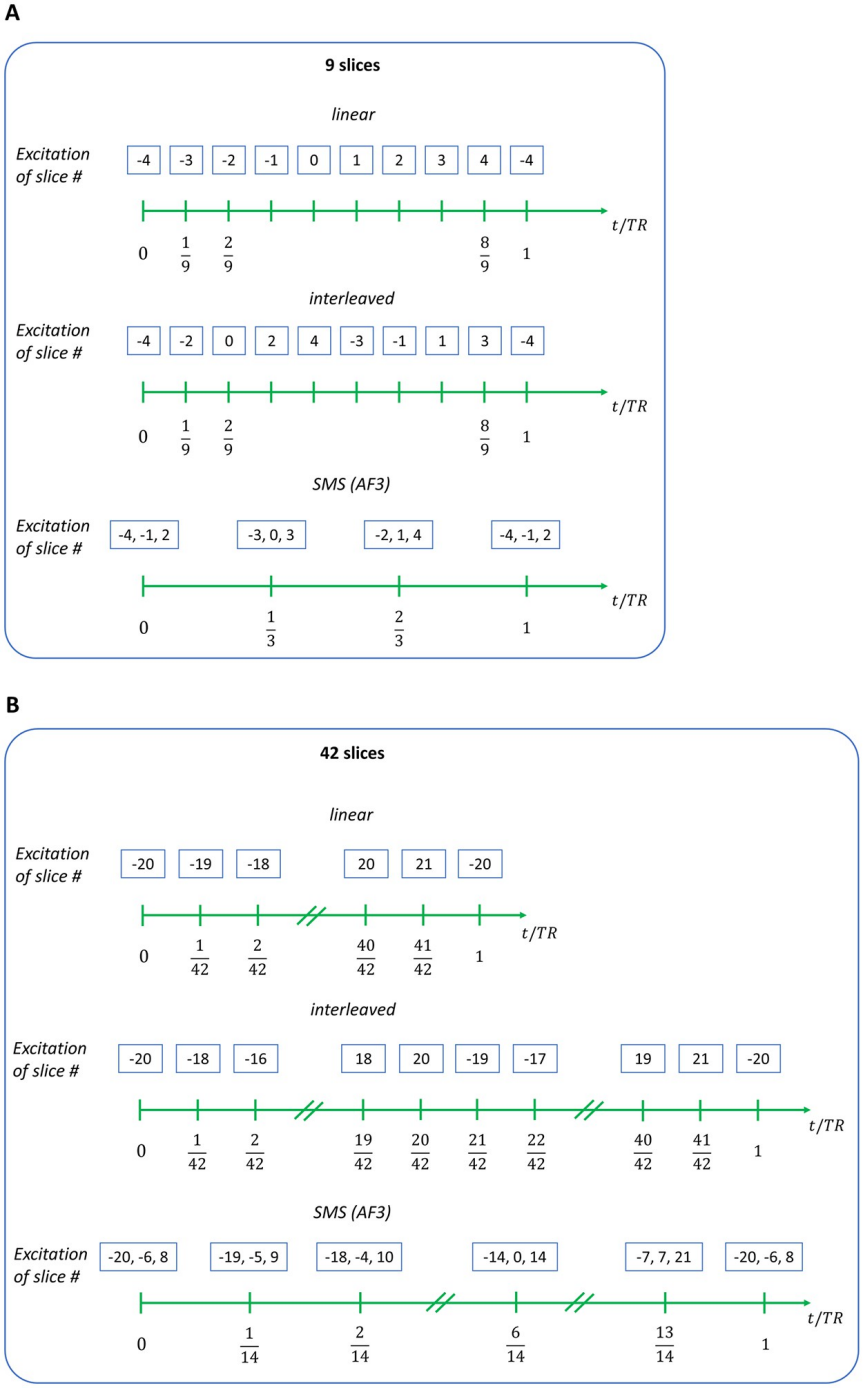
An illustration of the timing of slice excitations for the linear, interleaved, and SMS (AF3) acquisition modes that were determined via in-silico computations. Two settings with a total number of (A) nine and (B) 42 slices are depicted, respectively. AF: SMS acceleration factor.

A timing table of slice excitations was computed as illustrated in Fig 1, Tables 2 and 3. The slice excitation mode can be categorized into linear, interleaved, and SMS with AF = 3 (if the number of slices is divisible by three). The exact timing of the excitation pulse for each slice was determined based on these categories. We used the center slice (i.e., slice 0) to compute *f*.

**Table 2:** Timing table of slice excitations and propagation time *t*_initial slice_→0 for the linear, interleaved, and SMS (AF3) acquisition mode for a total of nine slices used in the in-silico simulations.

	Timing of excitation (1/TR)			*t*_initial slice→0_ (1/TR)		
*Slice #*	*linear*	*interleaved*	*SMS (AF3)*	*linear*	*interleaved*	*SMS (AF3)*
**-4**	0	0	0	49	29	13
**-3**	19	59	13	39	69	1
**-2**	29	19	23	29	19	23
**-1**	39	69	0	19	59	13
**0**	49	29	13	1	1	1
**1**	59	79	23	89	49	23
**2**	69	39	0	79	89	13
**3**	79	89	13	69	39	1
**4**	89	49	23	59	79	23

Slice 0 denotes the slice of interest in the simulations and is regarded as the center slice. Numbering starts at the top slice (negative) and increases toward the bottom slice.

**Table 3:** Timing table of slice excitations and propagation times *t*_initial slice→0_ for linear, interleaved, and SMS (AF3) acquisition mode for a total of 42 slices used in the in-silico simulations.

	Timing of excitation (1/TR)			*t*_initial slice→0_ (1/TR)		
*Slice #*	*linear*	*interleaved*	*SMS (AF3)*	*linear*	*interleaved*	*SMS (AF3)*
**-20**	0	0	0	2042	1042	614
**-19**	142	2142	114	1942	3142	514
**-18**	242	142	214	1842	942	414
**-17**	342	2242	314	1742	3042	314
**18**	3842	1942	1014	2442	3342	1014
**19**	3942	4042	1114	2342	1242	914
**20**	4042	2042	1214	2242	3242	814
**21**	4142	4142	1314	2142	1142	714

Slice 0 denotes the slice of interest in the simulations and is regarded as the center slice. Numbering starts at the top slice (negative) and increases to the bottom. Only eight representative slices are depicted here.

**Fig 2:**
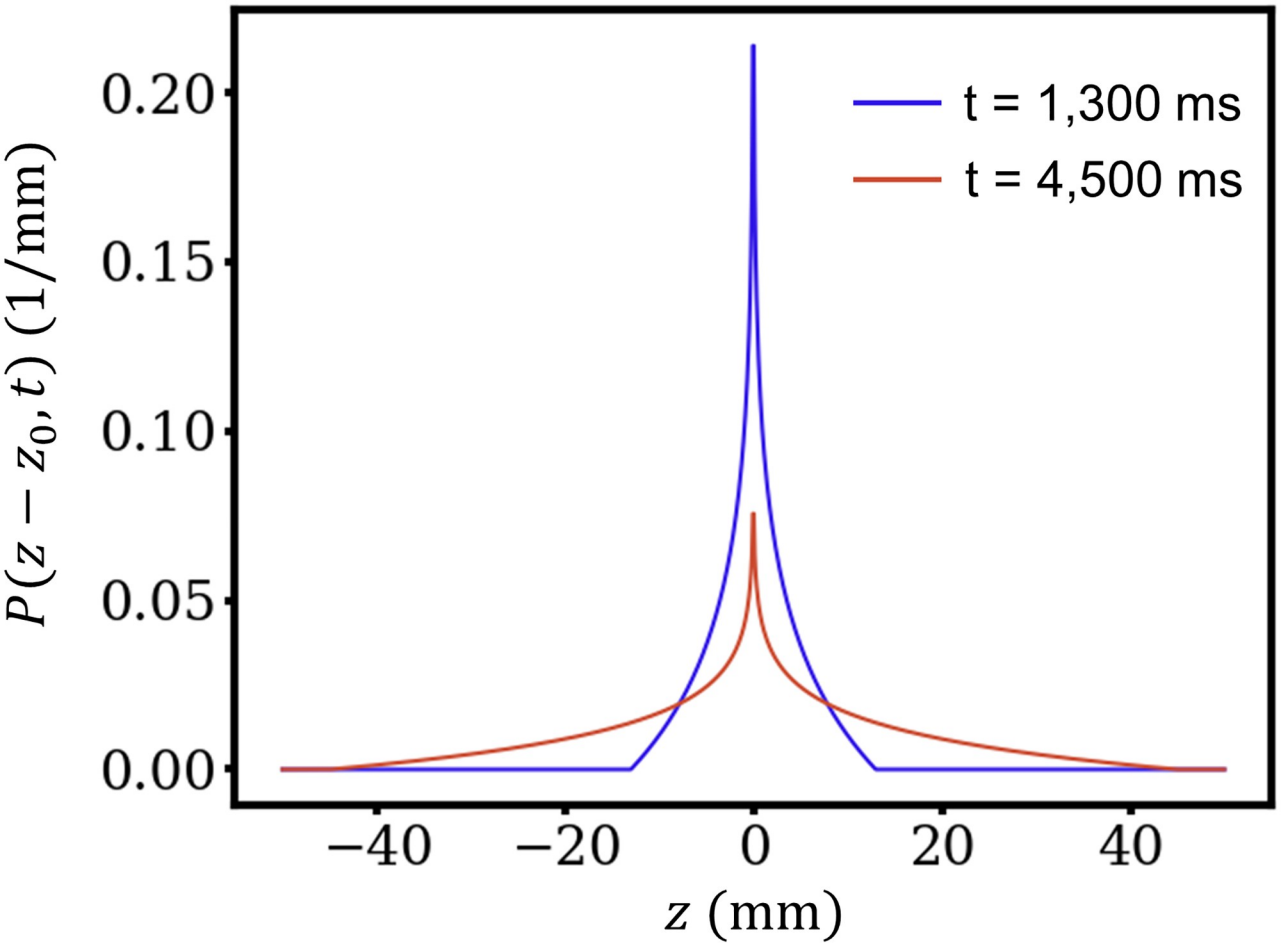
Propagator for laminar isotropic pipe flow for *z*_0_ = 0 and *v*_0_ = 5 mm/s with propagation times of 1,300 ms (blue) and 4,500 ms (red).

The propagator $P\left(\Delta z,t\right)=\frac{1}{4v_{0}t}ln\left(\frac{2v_{0}}{\left|\frac{\Delta z}{t}\right|}\right)\Pi \left(\frac{\Delta z}{t},-2v_{0},2v_{0}\right)$, which describes laminar isotropic pipe flow [6], was used to account for blood flow. The maximum absolute velocity perpendicular to the slice orientation is 2*v*_0_. $\Pi \left(\frac{\Delta z}{t},-2v_{0},2v_{0}\right)$ is the boxcar function, which is equal to 1 when $-2v_{0}\leq \frac{\Delta z}{t}<2v_{0}$ and 0 otherwise. Here, Δ*z* is the displacement in slice direction, and *t* is the propagation time. It should be noted that the propagator is normalized; i.e.,$\int_{-\infty }^{\infty }P\left(\Delta z,t\right)d\left(\Delta z\right)=1$. Fig 2 shows a representative propagator of the model for a laminar isotropic pipe flow of *v*_0_ = 5 mm/s with propagation times of 1,300 ms and 4,500 ms. Notably, the probability density is more dispersed for longer flowing times.

Equidistantly spaced sets of initial (*z*_initial_) and final positions (*z*_final_) were considered. The initial and final positions were spaced by 0.1 mm. The initial positions ranged from −42.5 mm to + 42.5 mm for the setting with nine 5 mm-thick slices and 5 mm gaps; from −30 mm to +55 mm for the setting with four 10 mm-thick slices and 15 mm-slice gaps; and from −102.5 mm to + 107.5 mm for the setting with 42 slices without slice gaps. The final positions ranged from −slice thickness/2 to +slice thickness/2. The blood signal attenuation factor was computed as follows:

$$r=\frac{1}{{\text{c}}_{\text{normalization}}}\cdot \sum_{\text{all}\;z_{\text{final}}\;\text{in}\;\text{slice}\;0}\left(\sum_{\text{all}\;z_{\text{initial}}\;\text{within}\;\text{a}\;\text{gap}}P\left(z_{\text{final}}-z_{\text{initial}},TR\right)+\sum_{\text{all}\;z_{\text{initial}}\;\text{within}\;\text{a}\;\text{slice}}P\left(z_{\text{final}}-z_{\text{initial}},t_{\text{initial}\;\text{slice}\to 0}\right)\cdot \left(1-e^{-\frac{t_{\text{initial}\;\text{slice}\to 0}}{T_{1}}}\right)\right)$$ (3)

where *r* describes the attenuation of the blood signal due to T_1_ relaxation. Here, *t*_initial slice→0_ denotes the propagation time from the slice of origin to slice 0 (c.f. Tables 2 and 3). The normalization coefficient c_normalization_ was determined by computing the double sum without the relaxation-weighting factor $\left(1-e^{-\frac{t_{initial\;slice\to 0}}{T_{1}}}\right)$. In the sum over the gap positions, the propagation time was set to *TR*, which is the time between consecutive excitations, in which the blood can propagate into slice 0. Blood from gap positions is unsaturated and consequently remains unweighted. The terms *P*(*z*_final_ − *z*_initial_, *TR*) and *P*(*z*_final_ − *z*_initial_, *t*_initial slice→0_) reflect the blood propagation from the initial positions *z*_initial_ to final positions *z*_final_. In our computations, we chose the center slice (slice 0) as our slice of interest and hence we only considered blood propagation into slice 0. Thus, all final positions *z*_final_ are within slice 0. The label "z_final_ in slice 0” denotes all position within the slice of interest. The initial positions for blood propagation *z*_initial_ are arbitrary and thus the summation over *z*_initial_ is performed over all slices and gap positions.

*f* is defined as

$$f=\frac{Blood\;signal}{Total\;signal}=\frac{f_{\infty }\cdot r}{f_{\infty }\cdot r+\left(1-f_{\infty }\right)\cdot \left[1-\text{exp}\left(-\frac{TR}{T_{1,liver}}\right)\right]}$$ (4)

where *f*_∞_ is the value of *f* for *TR* → ∞.

We did not measure *f*_∞_ in our experimental setup and instead used a finite *TR* and therewith measured *f*. In order to use *f*_∞_ as an input variable in the in-silico simulations, we estimated the value of *f*_∞_ using Eq 5:

$$f_{\infty }=\frac{f\cdot \left[\text{exp}\left(-\frac{TR}{T_{1,liver}}\right)-1\right]}{f\cdot \left[\text{exp}\left(-\frac{TR}{T_{1,liver}}\right)-1+r\right]-r}$$ (5)

*f*_∞_ was estimated from Eq 5 by setting *f* = *f*_meas_, where *f*_meas_ is the value obtained in our measurements or the measurements by Van et al. [18] (depending on the respective slice setting) for the long TR without SMS. We opted for the study by Van et al. over other publications because they reported the strongest effect on *f*.

This estimate of *f*_∞_ was used to compute the values of *f* for the other settings (such as short TR and SMS) using Eq 3 with their respective numerically computed *r* values.

In the context of model parameters, the T_1_ times of blood at 3T vary across the literature [35–40], ranging from 1,310 ms [36] to 1,932 ms [35]. The model proposed in our previous study [34] was most consistent with the measurements for a T_1_ between 1,300 ms and 1,600 ms. For this reason, we chose to set T_1_ = 1,300 ms for this study, except for one simulation in which we used 1,900 ms as a comparison. T_1_ times of the liver used in the simulations was 810 ms, consistent with the values reported by Stanisz et al. [35] (812 ms) and De Bazelaire et al. [41] (809 ms).

In-silico computations were performed using a range of acquisition parameters. Slice excitation modes were either set to linear, SMS with AF = 3, or interleaved (c.f. Tables 2 and 3, and Fig 1). We used a TR of 1,300 ms and 4,500 ms to match our experimental setup; simulations using a TR of 2,300 ms and 6,900 ms were also used to match the acquisition settings used by Van et al. [18]. The number of slices was either set to nine (5 mm slice thickness) and four (10 mm slice thickness), as in our study, or to 42 as used by Van et al. [18]. For simulations that matched our settings, we used slice thicknesses of 5 mm with 5 mm slice gaps; simulations matching the setting used by Van et al. were set to 0.01 mm slice gaps. Additionally, a slice thickness of 10 mm with 15 mm slice gaps was simulated. All computations were performed in Python 3.9.

## Results

**Fig 3:**
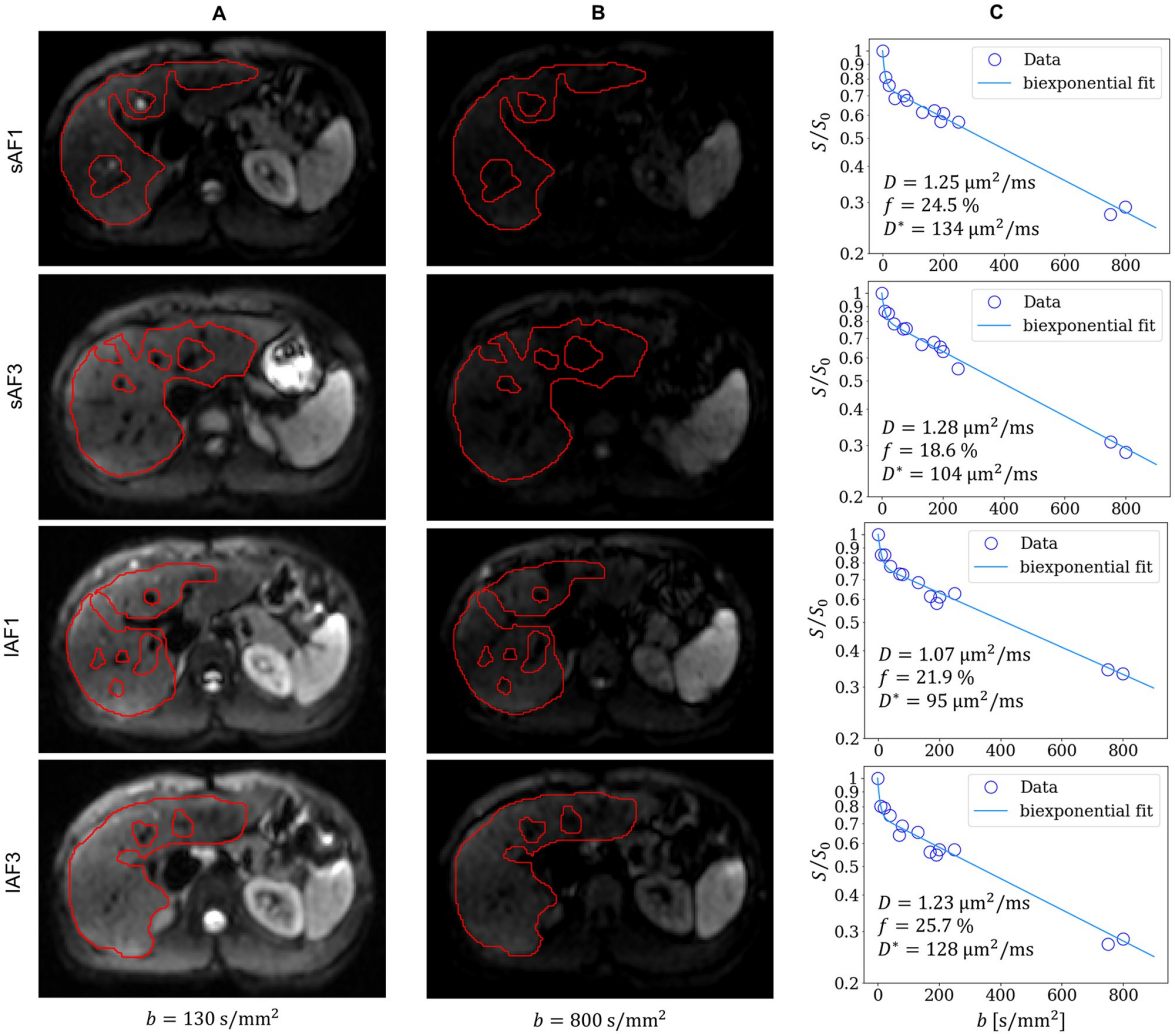
Representative diffusion-weighted images with ROIs (red) for sAF1, sAF3, lAF1, and lAF3 at (A) *b* = 130 s/mm^2^ and (B) *b* = 800 s/mm^2^, as well as (C) their respective biexponential IVIM fit curves.

Representative diffusion-weighted images for sAF1, sAF3, lAF1, and lAF3 at *b* = 130 s/mm^2^ and *b* = 800 s/mm^2^ with their respective ROI as well as their respective IVIM curves and biexponential fits are presented in Fig 3. The measured data are well described by the fitting curves.

**Fig 4:**
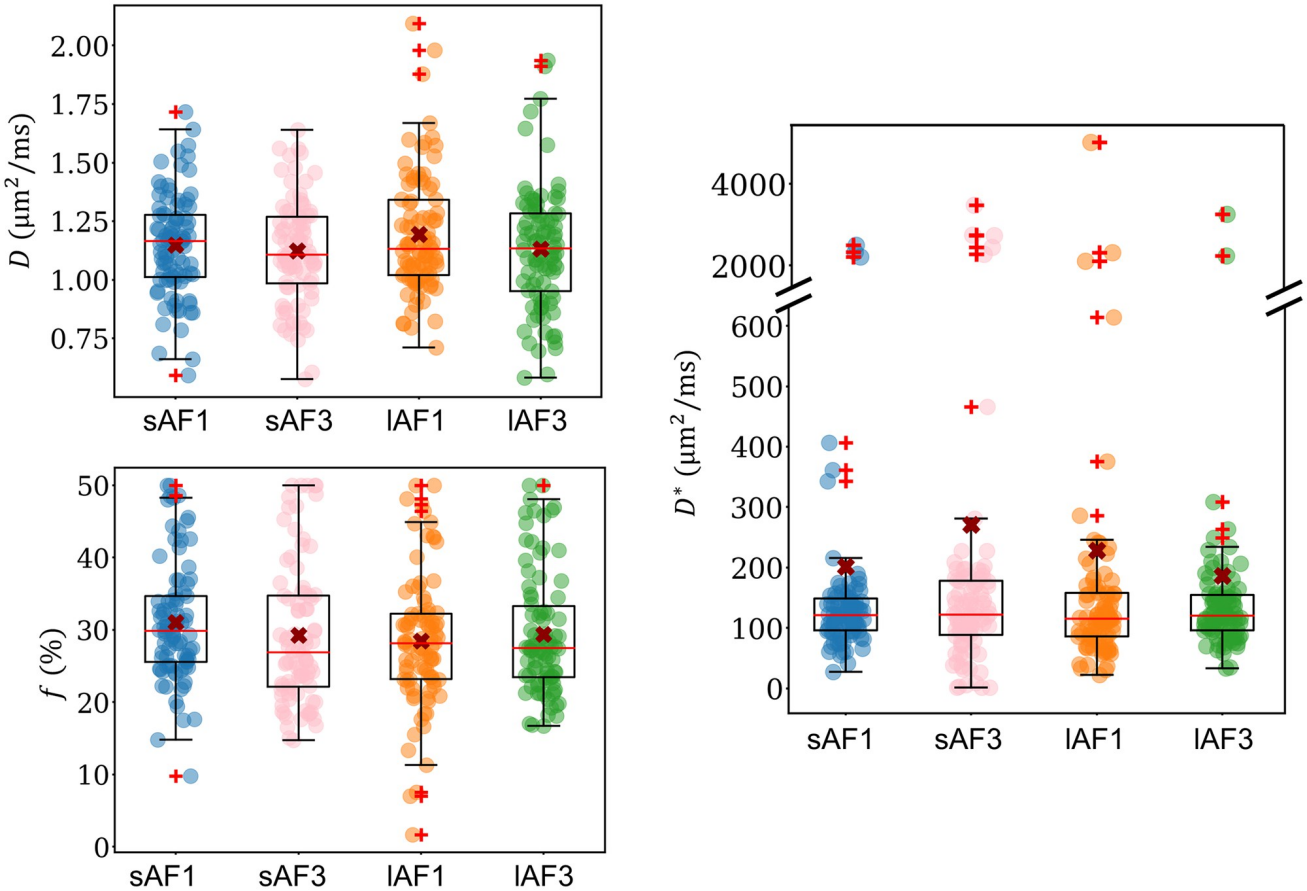
Boxplots of the biexponential IVIM parameters *D*, *f*, and D*. Each data point represents one slice. The median values are indicated by red lines, while the mean values are represented by red crosses. Outliers are marked using red ‘+’ signs. The interquartile range (IQR) is described by the edges of the boxes, while the whiskers indicate data within 1.5 ⋅ IQR. Note the discontinuous axis for D*. AF = SMS acceleration factor. TR for sAF1 and sAF3 = 1,300 ms; TR for lAF1 and lAF3 = 4,500 ms.

Boxplots showing the biexponential IVIM parameters for sAF1, sAF3, lAF1, and lAF3 are presented in Fig 4. The median values, first and third quartiles, and *p*-values are listed in Table 4. Predicted values of *f* for each of the different settings as derived from the simulations are also listed in Table 4. The results reveal that the differences in the IVIM parameters between the different settings are relatively small.

**Table 4:** The median, first and third quartiles of the biexponential IVIM parameters, as well as the calculated *p*-values and predictions of *f* as obtained from the simulations.

	*D* (μm^2^/ms)	*p*	*f* (%)	*p*	*f*_*sim*_ (%) (*v*_0_ = 2.5 mm/s)	D* (μm^2^/ms)	*p*
**sAF1**	1.16 [1.01, 1.28]	0.553 (Kruskal)	29.9 [25.6, 34.7]	0.122 (Kruskal)	27.8	121 [96, 149]	0.856 (Kruskal)
**sAF3**	1.11 [0.99, 1.27]		26.9 [22.2, 34.8]		26.7	122 [88, 178]	
**lAF1**	1.13 [1.02, 1.34]		28.1 [23.2, 32.2]		28.0	116 [86, 158]	
**lAF3**	1.13 [0.95, 1.28]		27.5 [23.4, 33.3]		27.9	120 [96, 154]	
**sAF1 (10 mm)**	1.18 [1.10, 1.37]	0.315 (t-test)	27.4 [22.2, 31.8]	0.141 (t-test)	26.8	99.1 [56, 151]	0.812 (Wilcoxon)
**lAF1 (10 mm)**	1.27 [1.06, 1.49]		31.3 [24.5, 34.6]		28.0	101 [61, 130]	

*p*-values were computed using the Kruskal-Wallis test, Student’s t-test, or Wilcoxon signed-rank test as indicated: Kruskal = Kruskal-Wallis test. t-test = Student’s t-test. Wilcoxon = Wilcoxon signed-rank test. AF = SMS acceleration factor. TR for sAF1 and sAF3 = 1,300 ms. TR for lAF1 and lAF3 = 4,500 ms.

The coefficients of variation (COV) for all measurement settings are reported in Table 5.

**Table 5:** Coefficients of variation (COV) for *D*, *f*, and D* for all measurement settings.

	*COV*_*D*_	*COV*_*f*_	*COV*_D*_
**sAF1**	0.136	0.250	0.529
**sAF3**	0.137	0.230	0.471
**lAF1**	0.106	0.267	0.208
**lAF3**	0.138	0.264	0.171
**sAF1 (10 mm)**	0.132	0.305	0.489
**lAF1 (10 mm)**	0.145	0.242	0.298

AF = SMS acceleration factor. TR for sAF1 and sAF3 = 1,300 ms; TR for lAF1 and lAF3 = 4,500 ms.

The median values of *D* were found to be relatively similar for sAF1, sAF3, lAF1, and lAF3. The *p*-value indicates that there was no significant difference between the median values (*p* = 0.553; Kruskal-Wallis test).

The Kruskal-Wallis test also revealed that there were no statistically significant differences in the *f* values of the four groups (*p* = 0.122). The median *f* in sAF3 was slightly smaller compared to sAF1, while the differences between conventional and SMS excitation were much smaller for long TR. The median *f* did not vary systematically between measurements at short and long TR: the median *f* was slightly higher for sAF1 compared to lAF1, and slightly lower for sAF3 compared to lAF3.

There were large uncertainties in the measurement of D* in all four groups; despite this, the median values of D* were very similar across the four acquisition settings tested, consistent with the results of the Kruskal-Wallis test (*p* = 0.856).

**Fig 5:**
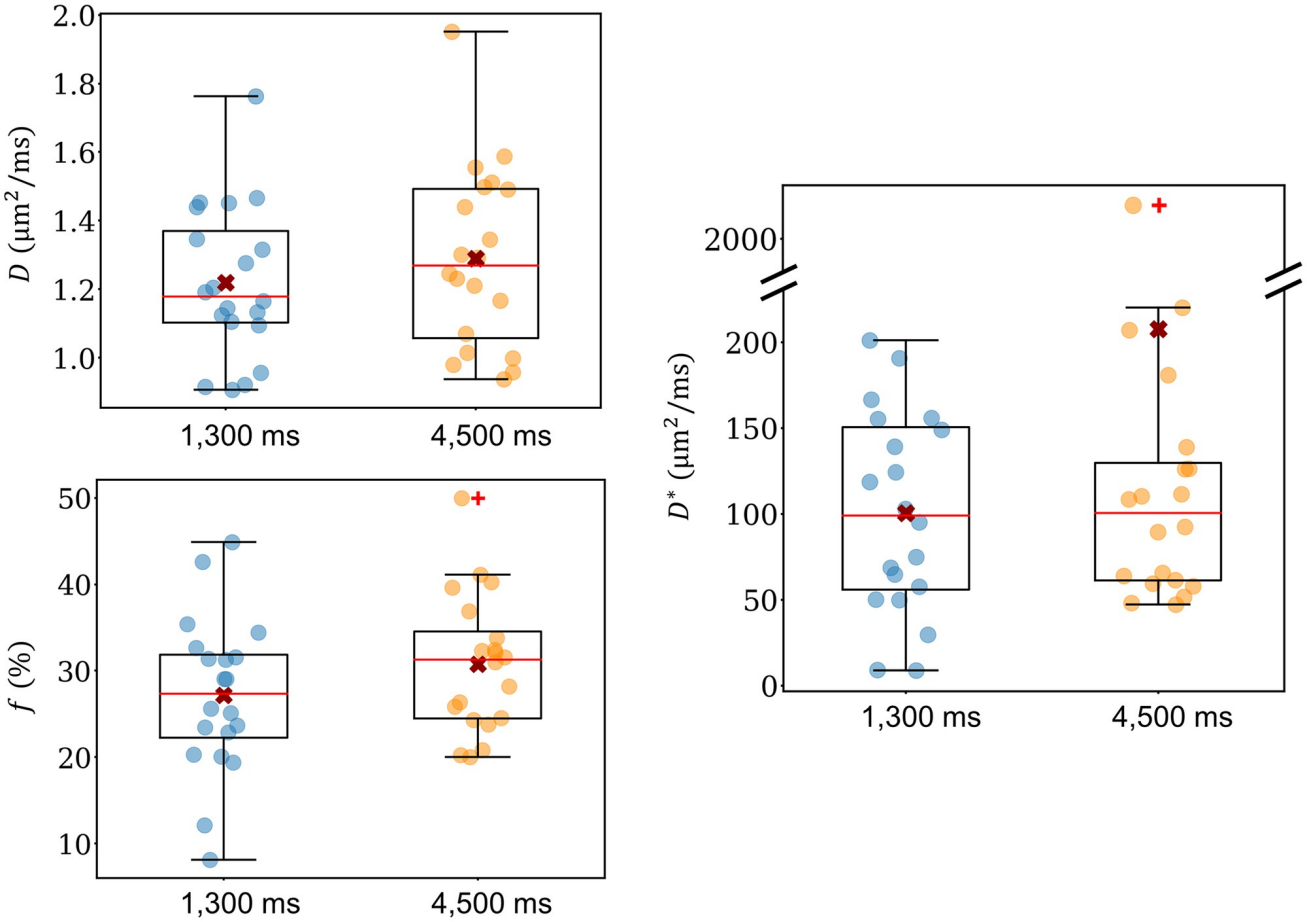
Boxplots of the biexponential IVIM parameters *D*, *f*, and D* for TR = 1,300 ms and TR = 4,500 ms collected using a slice thickness of 10 mm. Data were acquired in conventional slice excitation mode (AF1). Each data point represents one slice. Note the discontinuous axis for D*. AF = SMS acceleration factor.

Fig 5 shows the IVIM parameters for measurements conducted using 10 mm-thick slices with AF = 1. The median value of *D* at TR = 4,500 ms (1.27 μm^2^/ms) was slightly larger than obtained for TR = 1,300 ms (1.18 μm^2^/ms), but this difference was not significant (*p* = 0.315; Student’s t-test). Similarly, the value of *f* was reduced at the shorter TR acquisitions (27.4% compared to 31.3%) but not to a significant degree (*p* = 0.141; Student’s t-test). D* remained relatively equal across both protocols (*p* = 0.812; Wilcoxon). All median values, first and third quartiles, and *p*-values are presented in Table 4.

**Fig 6:**
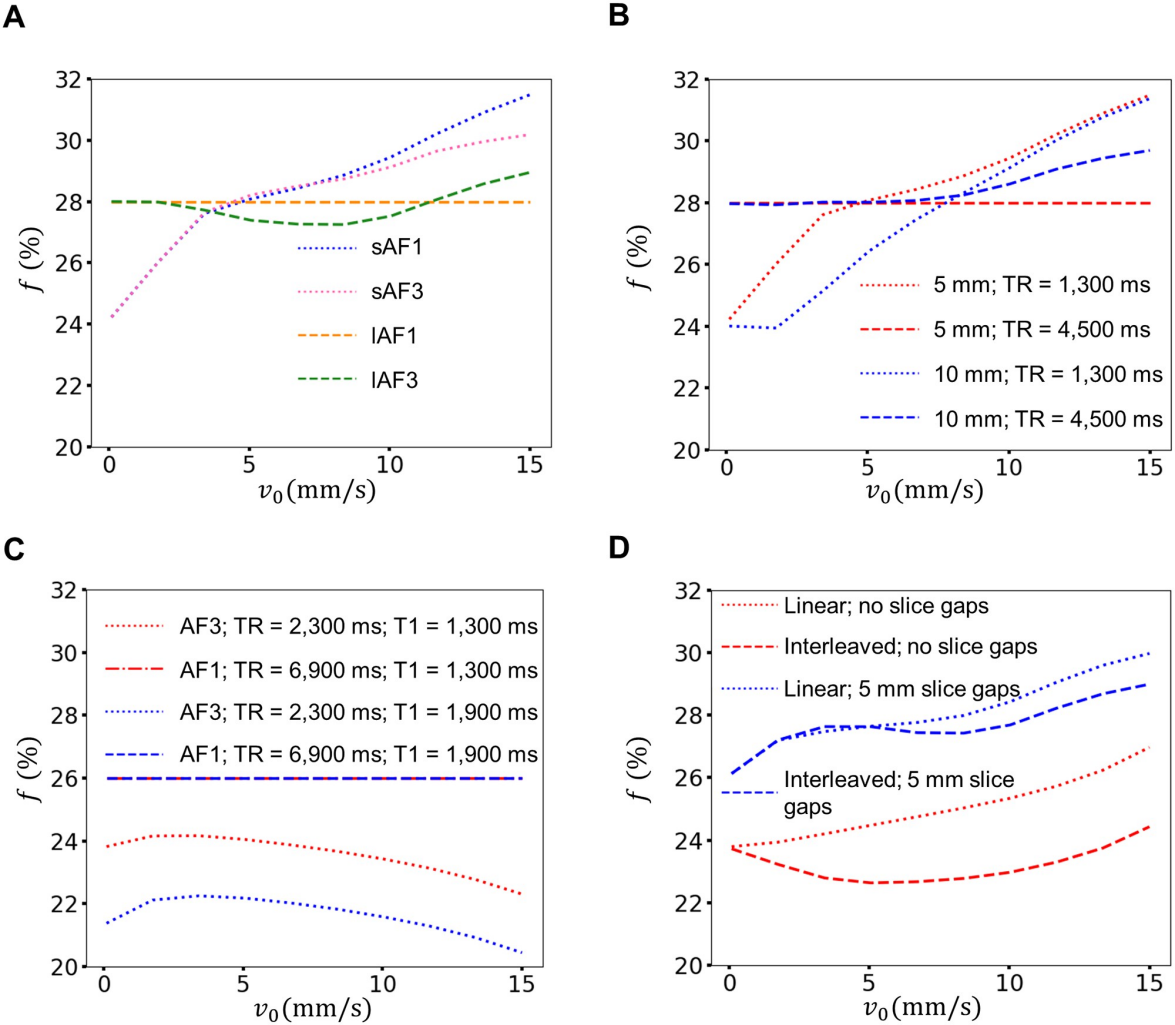
Predicted *f* values from the in-silico simulation for blood flow velocities *v*_0_ from 0 to 15 mm/s. (A) Comparison between sAF1, sAF3, lAF1 and lAF3. For *v*_0_ between 2.5 mm/s and 5 mm/s, there was no observable difference in *f* across acquisition schemes. (B) shows a larger TR-dependence of *f* for the 10 mm-thick slices with 15 mm spacing (blue) than for *f* in 5 mm-thick slices with 5 mm slice gaps (red). (C) *f* values using the acquisition parameters described by Van et al. [18] (42 slices with no slice gaps) at two T_1_ times (1,300 ms and 1,900 ms). The TR dependence of *f* is larger under these conditions than for the settings in (A) and (B). (D) The influence of interleaved acquisition mode on *f* at TR = 2,300 ms depends on the specific acquisition setting.

Fig 6 shows the predicted values of *f* according to the in-silico simulations conducted for different blood flow velocities *v*_0_. Fig 6A presents the predicted *f* for sAF1, sAF3, lAF1, and lAF3 with the respective acquisition parameters used in the measurements (i.e., 5 mm slice thickness with 5 mm slice gaps). The *f* value for lAF1 remains constant at 28% because it was used as the ground truth value for simulations (*f*_meas_). The predicted *f* for lAF3 remains nearly constant between 27–29%. *f* increases with *v*_0_ for sAF1 and sAF3, i.e., when TR = 1,300 ms. The simulation reveals that there is a certain range of blood flow velocities between 2.5–5 mm/s, where the modeled *f* values are very similar for all four acquisition settings. Fig 6B compares the modeled *f* values between our thin- and thick slice setups. The estimated *f* values for the 5 mm and 10 mm-thick slices were relatively similar, with *f* increasing with increasing *v*_0_ under short TR acquisitions. The T_1_-weighting is more pronounced for the 10 mm slice thickness. For example, at *v*_0_ ≈ 3 mm/s, *f* is equal for both short and long TR for acquisitions with 5 mm-thick slices, whereas *f* values from the 10 mm-thick slices exhibited clear differences between short and long TR (~25% and 28%, respectively). The *f* value at TR = 4,500 ms and 5 mm-thick slices serves as the ground truth value and is constant in the simulations. Predicted *f* values for the 10 mm slices at TR = 4,500 ms are constant at 28% until *v*_0_ = 5 mm/s, after which it increases with increasing velocity. Fig 6C presents simulations with acquisition parameters matching those described by Van et al. [18] (i.e., 42 slices with no slice gaps) for two T_1_ times (1,300 ms and 1,900 ms). The TR dependence of *f* is larger for the setting than those observed in Fig 6A and 6B. For TR = 6,900 ms, *f* remains constant at 26% (ground truth value). The short TR acquisition (2,300 ms) was modeled as an SMS acquisition with AF3; the *f* values were found to be smaller than long TR acquisitions and decreased from *v*_0_ ≈ 2 mm/s onwards. *f* was between 22–24% for T_1_ = 1,300 ms and between 20–22% for T_1_ = 1,900 ms. The interleaved slice mode was compared to linear slice acquisition (Fig 6D); it was found that the results depended on the specific slice setting. The two acquisition modes were compared under two regimes: (1) 42 slices with 5 mm slice thickness and negligible slice gaps and (2) nine slices with 5 mm slice thickness and 5 mm slice gaps; TR was 2,300 ms in both cases. The *f* values of the linear setting were consistently larger than in interleaved mode for 42 slices with no slice gaps at TR = 2,300 ms. In contrast, the difference between linear and interleaved is not as large when nine slices with 5 mm slice gaps were used. In particular, at *v*_0_ < 5 mm/s, the predicted values of *f* for linear and interleaved acquisition modes are almost equal, ranging between 26–28%. At higher *v*_0_, the dependence becomes slightly larger, but the difference remains smaller than 2%.

## Discussion

This study aimed to investigate the influence of SMS excitation and TR on the biexponential IVIM parameters. Previous studies reported differences between conventional slice excitation and SMS, especially with regard to *f* [17, 18, 20]. However, there is currently no detailed explanation for such differences in the literature. We assumed that secondary T_1_-effects due to reduced TR in SMS protocols were a potential explanation for this phenomenon; consequently, we performed examinations with and without SMS and with short and long TR to investigate this open issue.

Our study revealed no significant differences in the biexponential IVIM parameters obtained across these acquisition settings. This suggests that neither SMS nor TR had a significant effect on the IVIM parameters, at least for the acquisition setup used in this study. The missing effect on *f* at very short TR acquisitions was particularly surprising. Given a T_1_-relaxation time of blood between 1,300 ms [36] and 1,900 ms [35], the blood magnetization should be reduced to between 50–63% of the equilibrium magnetization at TR = 1,300 ms. However, it should be noted that the saturation of liver magnetization, as well as inflow and outflow effects, also play a role. For instance, De Bazelaire et al. [41] and Stanisz et al. [35] reported liver T_1_ times at 3T of 809 ms and 812 ms, respectively. This leads to a decrease of the liver magnetization to approximately 80% of the equilibrium liver magnetization for TR = 1,300 ms. To account for these effects, a more in-depth analysis was conducted using in-silico simulations described in the Methods section.

The simulations showed that the size of the saturation-induced effect is dependent on the blood flow velocity *v*_0_, the slice thickness, the size of the slice gaps, and the slice ordering. For blood flow velocities between 2.5–5 mm/s, the in-silico simulations were consistent with the experimental measurements of sAF1, sAF3, lAF1, and lAF3, exhibiting limited differences between the measured and predicted *f* values. This confirms that TR and SMS only have a marginal effect on *f* for the given acquisition parameters (i.e., 5 mm-thick slices with 5 mm slice gaps).

For thicker slices at low blood flow velocities, a higher fraction of saturated blood remains within the slice until its subsequent excitation, which may cause *f* to be considerably dependent on TR. The simulations were capable of reproducing these findings, with the values of *f* measured on 10 mm-thick slices exhibiting a stronger dependency on TR compared to the 5 mm-thick slices at flow velocities of *v*_0_ < 5 mm/s. Our previous study identified relevant flow velocities in the range of 1.5–2.5 mm/s [34]. Wetscherek et al. [42] found a mean blood flow velocity of 4.60 ± 0.34 mm/s in the liver using IVIM. Assuming that this blood flow velocity is solely attributable to capillary flow, these results are consistent with the literature, which reports capillary blood flow rates between 0.2 mm/s and 4 mm/s [4, 43, 44]. Consequently, we assume that all values below 5 mm/s fall within a physiologically reasonable range.

In general, the results of the in-silico investigations were consistent with the measured *f* values over the range of relevant blood flow velocities. Although the results were not completely consistent, this was to be expected from a relatively simple model that neglected, among other factors, the presence of different vessel sizes [45]. Indeed, we are aware of four previous studies in which IVIM was investigated using SMS techniques [17–20]. Van et al. [18] reported a significant decrease in *f* in the liver, kidney cortex, kidney medulla, pancreas, spleen, and erector spinae muscle with increasing slice acceleration factors. Specifically, during conventional excitation, the *f* in the liver was found to be 26%, 21%, and 15% for AF1, AF2, and AF3, respectively. Liver measurements conducted by Boss et al. [20] revealed a decrease in *f* from 16.6% (AF1) to 10.1% (AF3) in the first volunteer, but this trend was not observed for the second volunteer (17.2%, 24.9%, and 19.1% for AF1, AF2, and AF3, respectively). Tang et al. [17] reported that *f* decreased from 17% (AF1) to 11% (AF2) for benign lesions in the breast, but no significant reduction (15% and 14% for AF1 and AF2, respectively) was observed for malignant lesions. Xu et al. [19] reported similar *f* values for all three acceleration factors (26%, 25%, and 26% for AF1, AF2, and AF3, respectively) in the liver. Xu et al. also observed similar trends in *f* values in the pancreas, kidney cortex, kidney medulla, spleen, and the erector spinae muscle.

Our simulations indicate that the differences in the trends observed across these studies can be attributed to varying acquisition parameters. The relevant scanning parameters are listed in Table 6. Van et al. [18] reported the strongest effect size, stating that signal leakage in the slices [19] caused by the antialiasing of the SMS-DWI images may lead to inaccurate IVIM parameters. They also brought up potential T_1_-effects due to the reduction in TR. Their study did not use a fixed TR, but they exploited the main advantage of SMS acceleration and used significantly lower TR for their SMS accelerated data, with a TR of 6,900 ms, 3,500 ms, and 2,400 ms used for the accelerated protocols AF1, AF2, and AF3, respectively. Furthermore, unlike this study, Van et al. [18] used 42 axial slices within the liver, each with a thickness of 5 mm. Although they did not report the slice gap used, the number of slices and the slice thickness suggest that the slice gaps were either very small or zero, which would greatly increase the saturation of blood. The results of our simulations were somewhat consistent with this hypothesis (Fig 6C). Although the effect predicted by the model was not as large as that observed in Van et al. [18], the decrease in the simulated *f* from 26% to ~21% (T_1_ = 1,900 ms) and ~23% (T_1_ = 1,300 ms) from a TR of 6,900 ms to 2,400 ms suggests that this effect is significant. A similar effect may be present in the study conducted by Boss et al. [20], who, to the best of our knowledge, used the same protocol. The results of Tang et al. [17] must be interpreted with caution because, unlike the other studies, they did not examine healthy volunteers. They also significantly lowered the TR used for their AF2 measurements from 4,900 ms to 2,350 ms and used a slice thickness of 5 mm. However, the number and slice gaps were not reported. Finally, Xu et al. [19] performed measurements with 30 slices with a 6 mm slice thickness, suggesting that the slice gaps may have been larger than those used in the other studies discussed. This may explain why their measured *f* values did not exhibit any dependence on TR.

**Table 6:** Relevant imaging parameters of previous IVIM studies that utilized SMS techniques.

Study	TR (ms)			Slice Thickness (mm)	# Slices	Slice ordering	Slice gap
	AF1	AF2	AF3				
**Tang et al**. [17]	4,900	2,350	-	5	Not reported	Not reported	Not reported
**Van et al**. [18]	6,900	3,500	2,400	5	42	Not reported	Not reported
**Xu et al**. [19]	5,200	2,400	1,800	6	30	Not reported	Not reported
**Boss et al**. [20]	6,900	3,500	2,400	5	42	Not reported	Not reported
**This study**	1,300 4,500	-	1,300 4,500	5	9	linear	5 mm

TR = repetition time; AF = SMS acceleration factor.

Finally, our simulations revealed that any differences between linear and interleaved slice order modes are dependent on the specific acquisition setting. When using a setting with no slice gaps, the interleaved mode might lead to an effective reduction of TR due to the timing of slice excitations, resulting in smaller *f* values. For a setting with slice gaps, this effect is reduced due to the inflow of fresh blood magnetization from the slice gaps as well as the outflow of saturated blood magnetization into the slice gaps.

Our measurements, simulations, and the comparison between the aforementioned IVIM studies demonstrate that SMS very likely has no major influence on measured IVIM parameters. However, saturation effects are not always negligible and may have a substantial effect on measured *f* values. The size of this effect appears to depend on TR, the size of slice gaps, slice thickness, and slice order. While TR is regularly reported in imaging studies, slice gaps and/or slice order have often not been reported in past studies (including by authors involved in this study [9, 42, 46, 47]), as these parameters have historically been deemed to be of little relevance. Our conclusions suggest that this assumption is incorrect and that it should be best practice to report slice gaps and slice order in IVIM studies. Consensus-based recommendations may help avoid the issue of varying slice thicknesses (and of other acquisition parameters) among studies [48].

We acknowledge several limitations of our study. First, in order to keep TR constant across each measurement to allow for a straightforward comparison between IVIM parameters, images had to be acquired during free breathing. Unlike respiratory-triggered acquisitions, free breathing acquisition leads to reduced image quality, and, in the worst-case scenario, can generate motion artifacts. To address this issue, subjects were instructed to engage in shallow breathing to reduce any undesirable effects. In addition, several studies have reported free-breathing acquisition in IVIM in healthy volunteers to be preferable to respiratory/navigator triggering [49, 50], or at least state that there is no advantage to respiratory/navigator triggering during IVIM acquisition [51–53]. For instance, Jerome et al. [49] demonstrated a higher confidence in fitted IVIM parameters acquired during free-breathing due to fewer outliers. Similarly, Cieszanowski et al. [50] and Leporq et al. [52] reported on the possibility of using signal averages in IVIM during free-breathing and found that it led to enhanced reproducibility in IVIM parameters compared to respiratory-triggered acquisition.

Slice gaps were necessary to rule out the potential inflow of saturated magnetization from neighboring slices during the SMS acquisition, especially since the research questions in this study were focused on the influence of SMS. Although it also would have been interesting to investigate saturation effects with small slice gaps, their influence on IVIM parameters during conventional excitation has already been the topic of a previous study [34]. Slice gaps raise the issue of blind spots in the liver, i.e., areas with no measured information. However, our notion of the liver is that liver tissue is rather homogenous and that neither molecular water diffusion nor blood perfusion is expected to change significantly within slice gaps, at least when disregarding major vessels.

We are also aware of the assumptions and simplifications made for the in-silico simulations, such as the simplified model of velocity distribution (laminar isotropic pipe flow) that neglects the presence of different vessel sizes. We previously tested different propagators and found that they had little influence on the essential behavior of the model [34]. The simulations suggest that there is a physiologically and physically reasonable range in which the simulations can replicate the experimental measurements.

The evaluation in this study was “slice-wise”-based, meaning that each slice was evaluated separately. Consequently, there may be correlations between slices from the same volunteer that influence the results and the hypothesis testing. In the Supporting Information, an alternative approach based on a “slice-averaged” evaluation is presented (Section S1). In the slice-averaged method, median values are averaged over all slices for each volunteer and then fitted to the IVIM equation, effectively eliminating potential correlations between different slices from the same volunteer. This evaluation yields results very similar to those of the main manuscript.

Our smallest measured b*-*value was 10 s/mm^2^ (with the exception of *b* = 0). However, recent publications have revealed triexponential behavior at very low b*-*values [25, 54, 55]. Acquiring smaller b*-*values between 0–10 s/mm^2^ and using a triexponential fit would lower the fit uncertainty and may lead to more accurate results. However, the SMS-capable sequence used in this study only allowed for a step size of 10 s/mm^2^ at low b-values. Furthermore, a larger number of b-values would have increased the acquisition time of the experiment. In addition, triexponential behavior should not influence the measured *f*, the main target of our investigation; instead, it would have a greater effect on D*. Finally, though the sample size is similar to that of many other MRI research studies [56], examining more subjects would nevertheless have increased the statistical relevance of our data.

## Conclusion

In conclusion, the biexponential IVIM parameters measured in this study did not change significantly between SMS and conventional slice excitation. Small TRs also did not have any effect on measured *f* for a conventional slice thickness of 5 mm and sufficiently large slice gaps in liver DWI, although thicker slices could lead to a noticeable reduction in *f*. However, TR-related saturation effects can be quite substantial, as evidenced by differences in reported values of *f* in the literature. These differences presumably originate from T_1_-weighting and inflow effects due to a lowered TR and small slice gaps in slice accelerated SMS exams. In-silico investigations show that slice gaps and slice order may be of high importance and that it should be best practice to report these parameters.

## Supporting information

S1 File Additional validation based on a “slice-averaged” evaluation. (DOCX)

## Abbreviations

AF: SMS acceleration factor
ANOVA: one-way analysis of variance
DWI: diffusion-weighted imaging
IVIM: intravoxel incoherent motion
l: long TR (4,500 ms)
ROI: region of interest
s: short TR (1,300 ms)
SMS: simultaneous multislice
