# Supplementary material for: Effect of simultaneous multislice imaging, slice properties, and repetition time on the measured magnetic resonance biexponential intravoxel incoherent motion in the liver
Source: PLoS One. 2024 Aug 9;19(8):e0306996. doi: 10.1371/journal.pone.0306996 (PMC11315316; doi:10.1371/journal.pone.0306996)
Supplement: S1 File — (DOCX) [file pone.0306996.s001.docx]

# **Supporting Information**

## **Additional validation based on a “slice-averaged” evaluation**

In the following, IVIM parameters are determined by using a “slice-averaged” approach. After computing the median signal values and averaging over the diffusion directions and equal b-values, median values are computed over all slices for the same acquisition mode and volunteer instead of fitting data for each slice separately. In this way, potential correlations between slices of the same volunteer can be ruled out.

S1 Table shows all values for the median and first and third quartiles of the biexponential IVIM parameters and the results of the statistical analysis.

In S1 Fig, boxplots of the IVIM parameters for sAF1, sAF3, lAF1 and lAF3 are represented for the slice-averaged method and the “slice-wise” approach as performed in the main manuscript. Likewise, boxplots for the measurements with 10 mm slice thickness are depicted in S2 Fig.

The results for the slice-averaged evaluation method are similar to the slice-wise method. The spread of the data tends to be smaller for the slice-averaged approach, but median values are quite similar. The median of $f$ decreases from 29.5 % (TR = 4,500 ms) to 23.7 % (1,300 ms) for the 10 mm acquisition protocol. Yet, the statistical tests do not yield statistically significant differences for any of the given modalities similar to the slice-wise approach.

This comparison demonstrates the validity of the evaluation and statistical analysis in the main manuscript and indicates no dependency on the slice acquisition mode, but rather an influence of TR and slice thickness on $f$.

## **
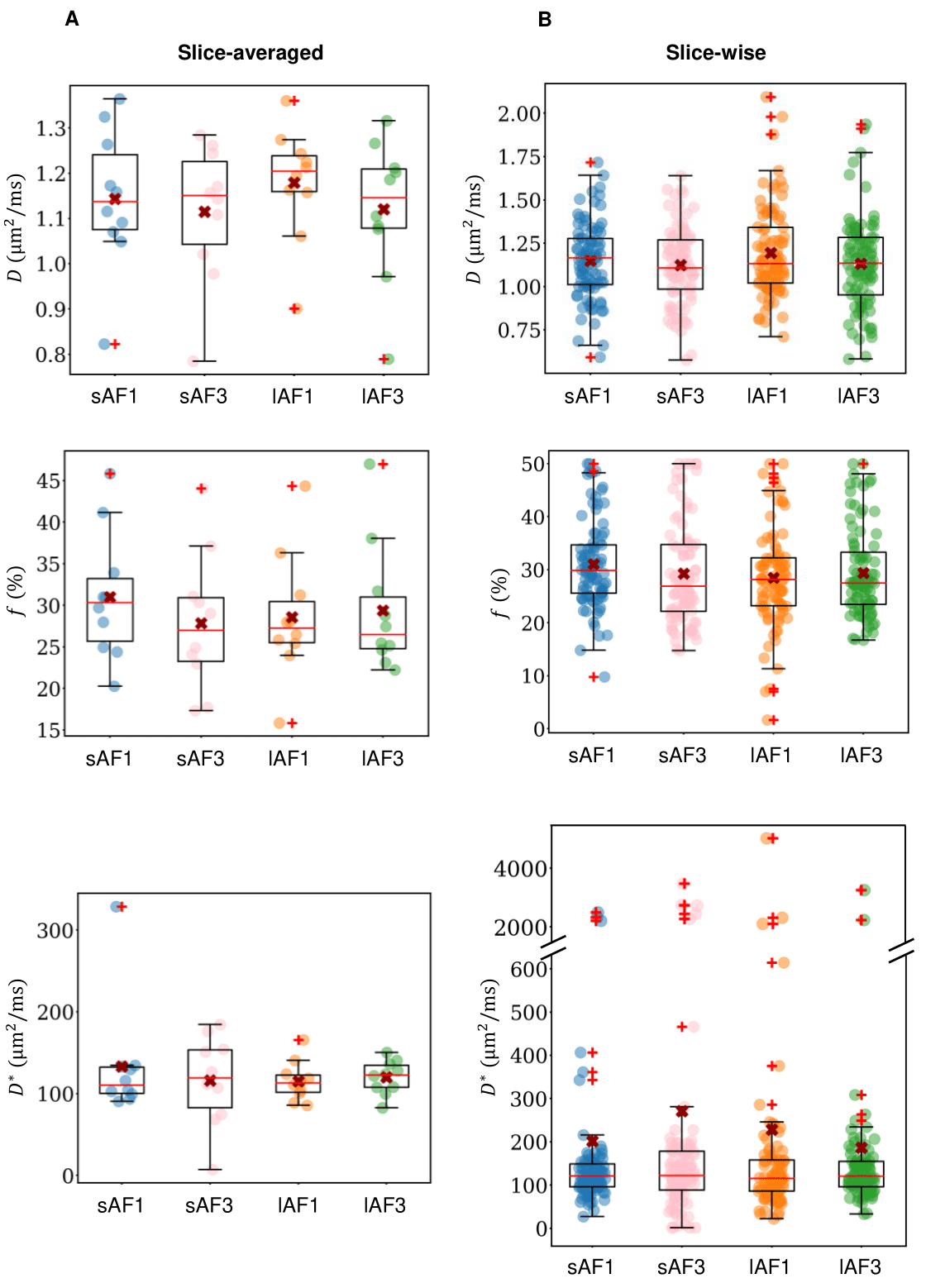
Figures**

**S1 Fig. Boxplots of the biexponential IVIM parameters** $\boldsymbol{D}$**,** $\boldsymbol{f}$**, and** $\boldsymbol{D}^{\boldsymbol{*}}$ **of the slice-averaged method (A) and the slice-wise method (B).** Each datapoint represents one volunteer in (A) and one slice in (B). The median values are indicated by the red lines, while the mean values are presented by red crosses. Outliers are marked using red ‘+’ signs. The IQR is described by the boxes, while the whiskers show data within 1.5 $\cdot$ IQR. AF = SMS acceleration factor. TR for sAF1 and sAF3 = 1,300 ms. TR for lAF1 and lAF3 = 4,500 ms.

**
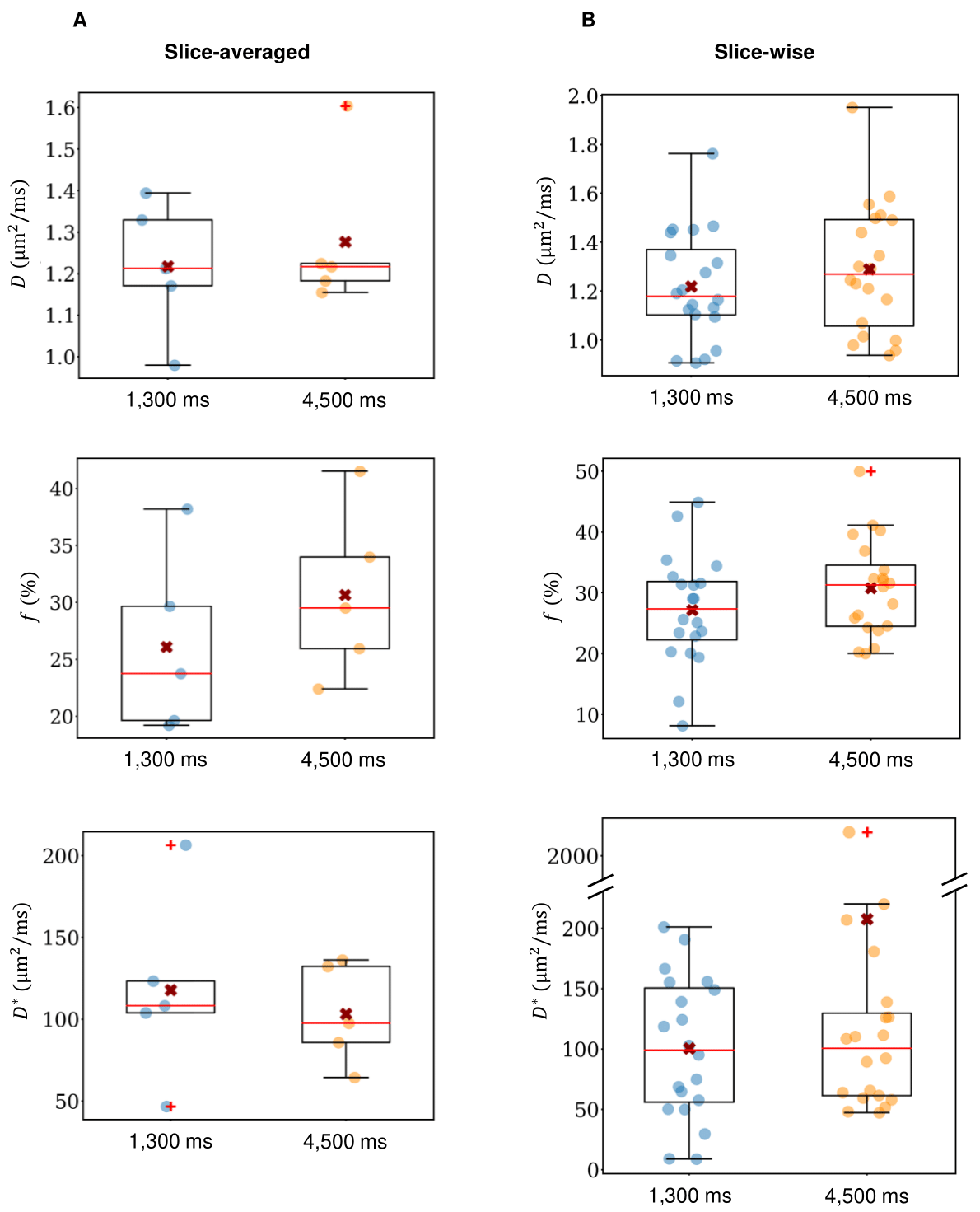
S2 Fig. Boxplots of the biexponential IVIM parameters** $\boldsymbol{D}$**,** $\boldsymbol{f}$**, and** $\boldsymbol{D}^{\boldsymbol{*}}$ **of the slice-averaged approach (A) and the slice-wise approach (B) for TR = 1,300 ms and TR = 4,500 ms.** Data was collected using a slice thickness of 10 mm and in conventional slice excitation mode (AF1). Each datapoint represents one volunteer in (A) and one slice in (B). Note the different axis scaling between (A) and (B). AF = SMS acceleration factor.

## **Tables**

|  | $\boldsymbol{D}$ (µm²/ms) | $\boldsymbol{p}$ | $\boldsymbol{f [\%]}$ | $\boldsymbol{p}$ | $\boldsymbol{D}^{\boldsymbol{*}}$ (µm²/ms) | $\boldsymbol{p}$ |
| --- | --- | --- | --- | --- | --- | --- |
| sAF1 | 1.14 [1.08, 1.24] | 0.761  (ANOVA) | 30.3 [25.7, 33.2] | 0.797  (Kruskal) | 110 [100, 132] | 0.935  (Kruskal) |
| sAF3 | 1.15 [1.04, 1.23] |  | 27.0 [23.2, 30.9] |  | 119 [83, 153] |  |
| lAF1 | 1.20 [1.16, 1.24] |  | 27.2 [25.5, 30.4] |  | 113 [101, 122] |  |
| lAF3 | 1.15 [1.08, 1.21] |  | 26.5 [24.8, 31.0] |  | 123 [107, 134] |  |
| sAF1 (10 mm) | 1.21 [1.17, 1.33] | 0.625  (Wilcoxon) | 23.7 [19.6, 29.7] | 0.289  (t-test) | 108 [104, 123] | 0.547  (t-test) |
| lAF1 (10 mm) | 1.22 [1.18, 1.22] |  | 29.5 [26.0, 34.0] |  | 97.5 [85.8, 132] |  |

**S1 Table. The median and first and third quartiles of the biexponential IVIM parameters, as well as the calculated *p*-values for the “slice-averaged” approach.** *p*-values were computed with the one-way ANOVA test, the Kruskal-Wallis test, Student’s t-test or Wilcoxon signed-rank test as indicated. AF = SMS acceleration factor. TR for sAF1 and sAF3 = 1,300 ms. TR for lAF1 and lAF3 = 4,500 ms. ANOVA = one-way ANOVA test. Kruskal = Kruskal-Wallis test. t-test = Student’s t-test. Wilcoxon = Wilcoxon signed-rank test.
